# The Third Meeting of the Iowa State Dental Society

**Published:** 1864-10

**Authors:** 


### Proceedings.

### THE THIRD MEETING OF THE IOWA STATE DENTAL SOCIETY.

**Held August 9th, 1864, at 8 P. M., at Drs. Robinson's and Trowbridge's Dental Rooms, Davenport, Iowa.**

After organization, Drs. Kulp and Sayles were appointed to receive Dr. Atkinson of New York, and Dr. Allport of Chicago, who were to arrive the next morning.

After some other business, Society adjourned till next morning, Aug. 10th, at 8 A. M.

SECOND DAY, 8 A. M.

Meeting called to order by Pres. Tulloss. Roll called and new members admitted. Eight new members signed the roll. On motion of Dr. Kulp, Drs. Atkinson and Allport were elected honorary members of the Society.

Filling teeth and sensitive dentine were the first subjects for discussion. The various methods of filling teeth with foil, and crystal gold, and tin, &c., were discussed at length, all present taking part, and on motion, the subject was continued until 3 P. M., and Drs. Atkinson and Allport were invited to give clinical demonstration before the society; the invitation was accepted.

SECOND—ALVEOLAR ABSCESS, CAUSES AND TREATMENT.

Dr. Henry S. Chase of Independence, opened the discussion by reading an Essay on this subject. Essay was placed into the hands of Publication Committee. Dr. Kulp explained his method of treating such cases, said he was always sure of a cure if he could reach the seat of disease with creosote—or creosote and iodine, said he generally treated through the tooth.

Dr. Atkinson was called on—he said he was greatly surprised to see that Iowa Dentists have arrived at such a high standard on this all important subject as was indicated by DR. CHASE'S Essay and DR. KULP'S remarks. It was a subject to which he had given a great deal of thought and attention, said he generally treated such cases by an incision from the outside of the gum—through process and bone down to seat of disease and then cut away all diseased bone and abscess, after which washed out the cavity with creosote and warm water, and thus continued to treat until the parts indicated a healthy condition—he said there was some danger of over treatment—he referred all to his published articles on this subject.

Dr. INGERSOLL, said—he never was heroic enough to cut through from the outside and treat in this way, said he generally treated through the tooth.

DR. ALLPORT, said he generally cut through and then took a burdrill and reamed out the old bone and cut the sack off of the root and treated with creosote and iodine—using warm water to syringe out with. The discussion continued with a great deal of interest and profit to all, and was participated in by all present.

On motion, the committee on popular education was called on for their report.

DR. KULP, chairman, made a verbal report—he said he had considered the propriety of publishing short articles in various branches profitable to the people, in Newspapers—but made up his mind that it was not the best plan—as it looked too much like advertising, and people would not read them, or if they did, they would feel that it was only advertising your business, and thus would not be profitable—he thought that if we adopted some such plan as the one on which the *People's Dental Journal* was gotten up on, we could succeed better—therefore, he thought we had better try and take all advertisements out of the *People's Dentist*, and put that in pamphelet form and bring it up to a high tone, and have that published under the auspices of the society, or if this is not possible, he suggested that we all try the *People's Dental Journal*—he said it could be gotten cheap enough to pay to distribute them among our patients—he thought it a good idea to have both on our tables for our patients to read. On this subject all had something to say.

Dr. Allport, was called on—he stated that he made some effort to educate the people on Dentistry in several ways, at his chair, and in the columns of the *Peoples' Journal.* Said that his connection with this journal was not for pecuniary benefit as he did not get one cent for his contributions or his efforts in keeping it up—said the publisher Dr. Haskell had the entire control of the publishing and subscription—therefore, he was the only one benefitted pecuniarly—he said he asked nothing for his "mite" in that direction, only what followed, knowing that his efforts were in the right direction.

On motion this committee was continued, and Drs. Robinson and Ingersoll were added thereon.

AFTERNOON MEETING.

After some business of society, Dr. Allport was furnished a patient for his clinical demonstration, the tooth was a first inferior molar on the left side, large cavity on grinding surface, his demonstrations and explanations in each step, were very interesting and instructive to all present—he filled the tooth with gold (of which he used nearly one eight ounce, by mallet pressure, his manner of keeping cavity dry for so long a time by the use of napkins was of profit to many present. The patient (our worthy landlord Dr. Burtis—of the Burtis House) was well pleased with his perfectly filled tooth, and expressed his entire satisfaction with the mallet, thought it a grand improvement on the old hand pressure, and as he is an old ex-dentist we must give his judgement some credit.

Dr. Atkinson operated on our worthy Secretary's teeth, filling two approximal cavities, between the left superior bicuspids, he astonished some of our Dentists by his heroic manner of using the wedge in separating teeth, "driving her home until the mallet rebounds," as he says, his demonstration put the "cap sheaf" in the superiority of the mallet and hand pressure, and most of the Iowa Dentists will undoubtedly use the mallet hereafter in filling teeth, we'll report at our next meeting.

The evening was occupied by holding a public meeting at Laclaire Hall, which was addressed by DRS. ATKINSON and ALLPORT on popular ignorance of Dentistry and the Dental organization, their hints were most capital and timely, and no doubt will do great good not only to the profession, but to all the people as they shall hear more of the same kind hereafter. A unanimous vote of thanks was passed to Drs. A. and A., for their address and their visit to our society—may they long live and come again. (Cor. Sec'ty.)

THIRD DAY—Met at 8 A. M., President in the Chiar.

1st. After usual business of organization the next subject was taken up, viz.: Deciduous teeth and Irregularities.

DR. KULP, thought this one of the most important subjects we have for consideration—he thought as a profession we did not give enough attention to deciduous teeth, parents and those having control of children did not know enough on the subject, and we as a profession are to educate them to a correct standard—we must therefore lay aside all our petty jealousies and go to work—if we do not, the public in many communities will get ahead of *us.* We can now see the want of knowledge of many of our profession, and especially of the Medical Profession in all communities on this subject by living examples. We must take more notice of these matters. In his opinion the best way to regulate irregularities in the future was to see properly to the deciduous teeth, where irregularities already exist—he thought that each dentist must be his own judge as to the mode of regulating.

DR. SAYLES, of Lyons, and DR. MAGILL, of Rockland, Ills., gave some diagrams of springs used by them in regulating teeth. It was generally conceded that if there was more attention paid to deciduous teeth there would be fewer cases of irregularities and less other dental derangement.

Extracting teeth was the next subject in order. All had something to say on this subject, and all regretted that there were so many teeth that had to be thus treated, the merits of different instruments were discussed at length.

Professional Etiquette was the next subject, a committee on this subject made the following report, which was accepted and passed into the hands of the Publication Committee:

REPORT.

The Committee on Dental Etiquette would respectfully offer the following hints for proper professional intercourse among the members of the Iowa State Dental Society.

The first duty of every member shall be, and is, to act up to the GOLDEN RULE "*Do unto others, even as ye would they should do unto you.*" Obedience to this command, would render further articles unnecessary.

But to be more explicit.

We consider it ungentlemanly, dishonorable, and degrading to the profession, as well as unjust to the people, for one of its members to endeavor to gain practice, by a reduction of the price of his operations below that of his neighbors, or of those with whom he comes in competition: for the effect of such conduct is to create an inferior class of operations, and lower the high standard of a profession which we are bound in honor to sustain.

It is the lowest depth of meanness to endeavor to seduce another patient to become your own, by an offer of services at a reduced price; or by inuendoes or hints of his incapacity.

The practice of "puffing" one's self into notoriety by drawing unfavorable comparisons between one's own professional ability and that of others, is ungentlemanly, unchristian, and should meet the frown of every true Dentist.

It *is* proper that Dentists should advertise their business in a quiet unassuming manner; which they *know* will not offend the good sense or delicacy of other members of the profession.

Those locating in a new town can very properly make known their place of business, and satisfy the public, by references, of their social, moral, and professional respectability.

We consider it reprehensible for a Dentist to interfere with the practice of another in towns evidently too small for the support of more than one permanent practitioner.

The custom of introducing circulars, and advertisements of that character beyond one's own legitimate sphere of practice, and where it would interfere with that of another, is much to be condemned.

Let each and every one of us assiduously watch our own conduct, and endeavor to behave both in word and deed as moral, upright gentlemen should, and as members of the same honorable profession.

H. D. BROWNSON,

HENRY S. CHASE, } *Committee.*

J. J. SEVERANCE.

Miscellaneous subjects was next in order.

Committee on Dental Fees reported a Fee bill with a suggestion that this society recommend it to the profession in Iowa, earnestly asking, all members to raise their prices to its standard and report at our next meeting, which was received and ordered as suggested by committee.

DR. INGERSOLL read an Essay on this subject which was put in the hands of the Publication Committee, with request that it be published in the *People's Dental Journal*, also that they have extra copies published for distribution among the profession and people.

The following Preamble and Resolutions were unanimously passed, and ordered to be published with fee bill.

WHEREAS, There has existed among Dentists a practice which has been well nigh universal, of competing with each other on the basis of low prices, and believing that in so doing there is a tendency to a depreciation of the character and quality of all Dental operations, detrimental to the interests of our patrons, and to the compromising of the honor and moral honesty of the profession, therefore

RESOLVED, That we declare ourselves ashamed of the low professional standard we have raised, and hereby pledge to each other and to the profession throughout the State, that we will cease all dishonorable competition and adopt in our practice only such measures of success as will be recognised by high-minded men of every profession as honorable and becoming the dignity of men and professional gentlemen.

RESOLVED, That the Iowa State Dental Society recommend the following fee bill to all respectable Dentists of our State and those adjoining, with the request that they raise their prices to its standard, and report at our next meeting:

| | |
|---|---|
| **OPERATIVE DENTISTRY.** | |
| Filling with Gold, smallest crown cavity, | $1.50 |
| " " " " approximal cavity | 2.00 |
| Larger and more difficult cavities and those requiring treatment, charge according to the requirements of the case. | |
| Smallest Filling with other material than Gold | 1.00 to $ 2.50 |
| Extracting teeth, | 50 to 1.00 |
| Administering Ether or Chloroform—extra, | 2.00 to |
| Scaling and Polishing, | 1.00 to 3.00 |
| **MECHANICAL DENTISTRY.** | |
| *Rubber Work.*—Full Upper and Lower Sets, on Rubber, | $50.00 to $75.00 |
| Intermediate charges varying according to the style, quality and completeness of the work. | |
| Full Upper Set, Rubber, Plain Teeth, | 25.00 |
| Best " " " Gum Teeth, | 40.00 |
| Single Tooth, | 8.00 |
| For Additional Teeth—each, | 2.00 |
| Teeth separated by a natural Tooth or Teeth intervening—each separation, | 1.00 |
| *Gold Work.*—Full Upper and Lower Set, | $150.00 to $200.00 |
| Full Upper Set, | 75.00 to 100.00 |
| One Tooth, | 10.00 |
| Each additional Tooth, | 3.00 to ——— |
| Continuous Gum, Full Set, | 200.00 |
| *Gold and Rubber Work.*—Gold and Rubber Combination, Full Set, | $150.00 to $175.00 |
| Partial Sets same as Gold Plate, | |

The following Resolution was passed.

*Resolved*, That all Dentists are requested to bring models of cases of irregularities, and of different cases of filling, also of other cases of interest to the next meeting.

The following was offered by DR. KULP, and ordered to be printed with the proceedings.

We have examined a drill stock invented by DR. WM. H. ROBINSON of Davenport, Iowa, and pronounce it a useful article, and ought to be in every Dentist's operating case, we, therefore, invite Dr. Robinson to put his design into the hands of some instrument manufacturer, so that the profession may have the benefit of it.

On motion of Dr. Ingersoll, the drill stock be named by the Iowa Dental Society—Robinson's Drill Stock.

Dr. Kulp was called on to give a report of his late trip to the National Association and "American Dental Convention." He gave a minute description of several improvements he saw in Dental offices, particularly of a very fine and handy operating case in the office of Dr. J. C. Dean of Chicago—and as Dr. Dean gave him the privilege to examine it with a view to having one manufactured for himself, he gave the Society the full benefit of his examination.

Quite a spirited discussion took place as to the best means of advancing our profession and especially of getting the profession more interested in our own State. It was finally decided, that our State Society must assume a Missionary character and go *to Dentists* if they do not *come* to *it.*

On motion, Des Moines, (our State Capital) was selected as the place, and the 2d Wednesday of January, 1865, at 9 A. M., the time, for holding our next meeting, at which the profession, in our State especially, are invited to be present.

Wm. O. Kulp,<br>

J. J. Severance, } *Publication Committee.*<br>

Wm. H. Robinson,

